# Mortality and Antibiotic Timing in Deep Learning-Derived Surviving Sepsis Campaign Risk Groups: A Multicenter Study

**DOI:** 10.21203/rs.3.rs-6123541/v1

**Published:** 2025-04-01

**Authors:** Ben J. Gross, Allison Donahue, James S. Ford, Xiaolei Lu, Aaron Boussina, Atul Malhotra, Kai Zheng, Shamim Nemati, Gabriel Wardi

## Abstract

**Background:** The current Surviving Sepsis Campaign (SSC) guidelines provide recommendations on timing of administering antibiotics in sepsis patients based on probability of sepsis and presence of shock. However, there have been minimal efforts to stratify patients objectively into these groups and describe patient outcomes as a function of antibiotic timing recommendations based on risk stratification using this approach. **Methods:** We conducted an observational cohort study using prospectively applied patient data from two large health systems using patient encounters between 2016 and 2024. At the time of clinical suspicion of sepsis, two deep learning (DL) models were used to stratify patients objectively into groups analogous to the SSC risk groups, based on a patient’s likelihood of having sepsis and likelihood of developing shock. These risk groups were: 1) shock likely to develop and sepsis probable, 2) shock likely to develop and sepsis possible, 3) shock unlikely to develop and sepsis probable, and 4) shock unlikely to develop and sepsis possible. The primary outcome was short-term mortality, a composite of in-hospital mortality and transition to hospice care, across each risk group. **Results:** We identified 34,163 adult patients with potential sepsis. At the development site, risk group mortality rates (%) and median time to antibiotics [IQR] were as follows: 1) 23.1%, 1.7 [1.0 - 3.1] hours; 17.7%, 3.0 [1.7 - 6.2] hours; 5.0%, 2.8 [1.5 - 5.1] hours; and 1.9%, 4.6 [2.7 - 8.0] hours. Results from the validation site were similar. Mortality rates were similar for patients with probable sepsis unlikely to develop shock regardless of antibiotic administration within 1 or 3 hours from triage. **Conclusions** : Our results suggest that patients who are at low risk of developing shock, regardless of their likelihood of having sepsis, had similar rates of mortality in the 1-hour vs 3-hour time to antibiotic administration groups. Thus, a more lenient time to antibiotic administration could allow for more detailed evaluations and judicious administration of antibiotics, without impacting patient mortality, although we did not assess for causation. Additional prospective studies are required to validate these findings.

**Additional Declarations:** Competing interest reported. S.N., A. B., and A.M. are cofounders, advisors, and hold equity in Clairyon Inc. (formerly Healcisio Inc.), a medical software startup. The terms of this arrangement have been reviewed and approved by the UC San Diego in accordance with its conflict-of-interest policies. S.N. and A.M. report income from Powell Mansfield, a startup focused on diagnosis of sleep disordered breathing. A.M. additionally reports income related to medical education from Livanova, Eli Lilly, and Zoll. The remaining authors declare no competing interests.

## BACKGROUND

Sepsis, a dysregulated host response to infection, affects over 1.7 million people annually and is responsible for over 350,000 deaths each year in the United States (US) [1]. Sepsis also remains a major driver of healthcare costs, totaling over $62 billion in annual expenditures in the US [2]. In 2021, the Surviving Sepsis Campaign (SSC) updated its Guidelines for the Management of Sepsis and Septic Shock to recommend antibiotic timing (within 1 or 3 hours) based on severity of illness (presence or absence of shock) and probability of sepsis (“definite or probable” vs “possible”) [3] . This clinical construct produced four categories of risk. However, this approach is inherently subjective, and physicians may disagree about the probability of sepsis in many cases [4,5] .

The SSC provides a strong recommendation to administer antibiotics within 3 hours to patients with possible sepsis and within 1 hour to patients with probable or definite sepsis, or those with shock[3]. In the US, the Centers for Medicare and Medicaid (CMS) Severe Sepsis and Septic Shock Management Bundle (SEP-1) also provides strict recommendations regarding timing of antibiotics for patients with suspected or confirmed severe sepsis or septic shock[6]. Various professional societies have expressed concerns about rigid time periods for antibiotic administration, particularly when significant diagnostic uncertainty exists[7,8]. Indeed, in patients with possible sepsis but without shock, there are minimal moderate or high-quality data that suggest antibiotic administration within 3 hours improves patient-centered outcomes[9]. However, identifying which patients have sepsis and which patients will later develop shock can be difficult, and to our knowledge, no objective tool or model is available to assist in identifying these at-risk patients. Thus, we applied two deep learning (DL) models to determine a patient’s risk of 1) having sepsis (“possible” vs. “probable”), and 2) developing shock (“shock likely to develop” or “shock unlikely to develop”). The output of these two models produces four risk groups that are analogous to those described above by the SSC.

In this study, we sought to determine the association between timing of antibiotic therapy and short-term mortality in patients with clinical suspicion of sepsis based on the probability of sepsis and risk of developing shock. To accomplish this, we first utilized 2 DL models to partition patients with clinical suspicion of sepsis into the abovementioned SSC risk groups. Next, we stratified patients in these partitions based on timing of antibiotic administration from time of clinical suspicion of sepsis to determine associations with short term mortality. We hypothesized that mortality would differ as a function of antibiotic timing within each risk group and across risk groups.

## METHODS

Study Design and Setting:

We conducted a multicenter cohort study of Emergency Department (ED) patients across two large health systems. Our development site was the University of California, San Diego Health (UCSD), where we collected patient data from two hospital EDs between January 2016 to December 2023. One of UCSD’s hospitals serves as the “safety net” hospital in San Diego and the other is a quaternary care center with a combined annual ED census of 100,000 patients. Our validation site was the University of California, Irvine (UCI) where we collected patient data between January 2023 to August 2024. UCI has a single ED with an annual census of approximately 64,000 patients. Institutional review board approval with waiver of informed consent was obtained.

Study Population and Selection of Participants:

All adult patients (at least 18 years) who presented to the ED during the study period were automatically queried for clinical suspicion of sepsis using data available from the electronic health record (EHR). Our method of data abstraction was previously described elsewhere [10]. We included all adult ED patients who met criteria for clinical suspicion of sepsis within the first 24 hours of ED triage. We defined clinical suspicion of sepsis at the presence of 2 of 4 of the systemic inflammatory response syndrome (SIRS; heart rate > 90 beats per minute, respiratory rate > 20 breaths per minute, white blood cell count > 12 cells/uL or < 4 cells/uL or >10% bands or temperature > 38.3C or <36C) were present within 6 hours and the ordering of two of the following: intravenous antibiotics, blood cultures, or lactate within the first 24 hours from ED triage. The time of potential sepsis ("time zero") was defined as the time when the last of these criteria were met. We chose this approach and definition because it corresponds to the time when bedside providers would have had sufficient concern for sepsis to place sepsis-related orders. We excluded pediatric patients (age < 18 years old), patients transferred to other facilities during their hospitalization, encounters without any laboratory results or vital signs while in the ED (see Figures 1 & 2). As SSC guidelines recommend that patients with shock receive antibiotics within one hour, we excluded patients who were already in shock when the "clinical suspicion for sepsis" definition was met.

We used "Sepsis 3" definitions to define clinical sepsis and septic shock[11]. Within this construct, clinical sepsis was defined as a 2-point increase in a patient's baseline sequential organ failure assessment (SOFA) score *and* suspicion of infection (checking blood cultures and administering intravenous antimicrobial administration for at least 72 hours). The 2-point change in SOFA could occur either 24 hours prior to the suspicion of infection *or* up to 12 hours after the suspicion of infection. The onset of clinical sepsis ($t_{sepsis}$) was defined as a 2-point change in SOFA score or suspicion of infection, which ever came first. Septic shock was defined as the presence of sepsis and the initiation of a titratable vasoactive medication. As some patients did not have a serum lactate near the time of shock, we did not require a lactate of at least 2 mmol/L in our criteria for septic shock.

Variable Selection and Data Pre-Processing

Variables included patient demographic information, vital signs, laboratory test results, medications, and co-morbidities, among others (Supplemental Tables 3 & 4). Data missingness is reported in the Supplement. Features in the development cohort set underwent normality transformations and Z-score normalization (subtracting the mean and dividing by SD). All the testing and validation data sets were normalized with the mean and SD computed from the development cohort training set.

Sepsis Risk Groups

At the time of clinical suspicion of sepsis, two DL models were used to objectively stratify patients into groups analogous to the SSC risk groups, based on a patient's likelihood of having sepsis and likelihood of developing shock. These risk groups were: 1) shock likely to develop and sepsis probable, 2) shock likely to develop and sepsis possible, 3) shock unlikely to develop and sepsis probable, 4) shock unlikely to develop and sepsis possible.

Deep Learning Model Development

To determine the likelihood of sepsis, we used the COMPOSER (Conformal Multidimensional Projection of Sepsis Risk) model, which has been previously described[12]. COMPOSER was developed using patient encounters from UCSD and Emory University. It achieved a high area under the curve (AUC: 0.938–0.945) in the real-time prediction of sepsis in the ED and its implementation was associated with a 17% relative decrease in mortality in sepsis patients[13]. COMPOSER generates a score (between 0 and 11), where a higher score corresponds to an increased likelihood of sepsis. In the present study, patients with COMPOSER score of ≥6 were assigned to the SSC category “sepsis probable” and patients with a score <6 were assigned to the SSC category “sepsis possible.” The COMPOSER score was derived at the time of clinical suspicion of sepsis”.Our approach for determining “possible” and “probable” sepsis was derived from Taylor et al.[14], where “probable sepsis” refers to at least a 50% chance of higher of developing sepsis.

To predict the risk of developing shock we created Shock-Net, a transformer-based neural network. Shock-Net was based upon the previously described AISE model to predict shock in ED patients[10]. As with COMPOSER, the Shock-Net score was derived to provide predictions on the probability of a patient developing shock at the time of clinical suspicion of potential sepsis. Shock-Net generated a score (between 0 and 1), where a higher score corresponded to an increased likelihood of developing shock. Patients with Shock-Net scores <0.88 were assigned to the “shock unlikely to develop” group, whereas patients with scores ≥0.88 were assigned to the “shock likely to develop” groups. The Shock-Net model was trained on 62,846 patients in the ED, ICU, and general wards at UC San Diego, with a prediction window of 6 hours. The threshold above which the model predicts shock is determined from the model development validation, which we tune to be 60% sensitivity per patient.  It was validated on an additional 15,716 patients from these units. For validation the data were split 80/20 into training and testing sets. Shock-Net achieved an AUC of 0.959 during validation testing on UCSD patient data. It was further validated on 4776 ED patients at UC Irvine achieving an AUC of 93.7% on this external validation set.

Outcomes and Other Definitions:

Our primary outcome was a short-term mortality, a composite measure of in-hospital mortality or transition to hospice care, which is more complete representation of end-of-life destination than in-hospital mortality alone[15,16]. We stratified the primary outcome by SSC risk groups and by time-to-antibiotics (<1 hour vs <3 hours). Time-to-antibiotics was defined as the time from triage in the ED to administration of intravenous (IV) antibiotics. Patients were tagged as “clinical sepsis” (for ground truth labeling) if they met the Sepsis 3 criteria and “clinical shock” if vasoactive medications were initiated within 24 hours of ED triage.

Analysis:

In our primary analysis, we included all patients who met inclusion criteria to assess for probability of sepsis and shock development. We also performed a sub-analysis excluding patients who developed shock within the first 3 hours of ED triage. This sub-analysis was conducted because prior investigations have highlighted the importance of identification of patients with delayed septic shock after initial provider evaluation[17,18]. We chose the 3-hour time interval after triage as a reasonable time based on prior experience from the clinician investigators experience and published literature[10,17,18].

By sub-stratifying each risk group into those who received antibiotics within 1 hour of triage, within 1-3 hours of triage, and those who received antibiotics more than 3 hours after triage, we assessed whether early antibiotics significantly decreased mortality within each risk group and whether delayed antibiotics significantly increased mortality within each risk group. These hypotheses were tested using one-sided Pearson's Chi-square test for independence. First, we applied this Chi-squared test to see if the mortality of those who received antibiotics within an hour of triage in each risk group were significantly lower than the other patients within the risk group. Then we applied this Chi-squared test to see if those who received antibiotics after 3 hours of triage had significantly higher mortality compared to the other patients within the risk group. A p-value $< 0.05$ was considered significant for these one-sided Chi-squared tests.

We also report mortality rates and time-to-antibiotics using descriptive statistics for each risk group. Differences between groups and within groups were assessed using Kruskal-Wallis tests and Chi-squared tests, respectively. To reduce the risk of Type I error from assessing multiple pairwise comparisons, we used a Bonferroni correction to adjust the significance threshold. A p-value $< 0.05$ was considered significant in our overall comparisons, and to maintain this level of significance in making our 6 pair-wise comparisons between each group we adjust the significant threshold to $\alpha = 0.0083$ by dividing our significance threshold by the number of pairwise comparisons. Full statistical analyzes are reported in the Supplement. Both models were developed and processed using Tensorflow and Python 3. The SciPy stats package was used to perform all statistical analysis.

## RESULTS

### Patient Characteristics

We included 29,790 patients with clinical suspicion of potential sepsis at the development site and 4,373 patients at the validation site (Figs. 1). Full patient characteristics for the development and validation sites, stratified SSC risk groups, are reported in Tables 1 and 2.

### Antibiotic Timing

Among patients who received antibiotics at the development site, 48.4% (12124/25029) received them within 3 hours from triage, and 12.7% (3177/25029) received them within 1 hour from triage. At the validation site 39.6% (1588/4012) of patients who received antibiotics received them within 3 hours of triage and 13.2% (1588/4012) of these patients received them within 1 hour of triage. A detailed description of antibiotic timing among all risk groups can be found in Supplementary Tables 5 & 6.

### Short Term Mortality

Among all patients, short term mortality was 6.1% (1825/29790) and 9.3% (405/4373) at the development site and validation sites, respectively (Fig. 3).

At the training site, short term mortality was highest in the groups that were identified by Shock-net as having a high likelihood of developing shock (Group 1, 23.1%; Group 2, 17.7%). These two groups also had the shortest time to antibiotics (Group 1: 1.7 hours [IQR 1.0 to 3.1], Group 2: 3.0 hours [IQR 1.0 to 3.1]. Results were similar at the validation site. In patients who actually developed shock within 3 hours of triage, mortality was high at both the development site (31%, [470/1,534] and the validation site (40% [92/230]).

At both the training and validation sites, short term mortality was not significantly lower between groups that received antibiotics within 1 hour or after 1 hour (**Table 3**). Similarly, short term mortality was not significantly different in the groups that received antibiotics before or after 3 hours.

### Sub-analysis Excluding Patients Who Developed Shock Within 3 Hours of ED Triage

After excluding patients who had shock within 3 hours of ED triage, there were 28,256 patients and 4,143 patients at the development and validation sites, respectively (Fig. 2). In this sub-analysis, trends in time-to-antibiotics and mortality were similar overall (Fig. 4) and across risk groups (Table 4, Supplemental Tables 7 & 8).

### DISCUSSION

In this multicenter study using prospectively applied data from 34,163 patients, we have two major findings that may help inform timing of antibiotics in patients with potential sepsis. First, we demonstrate that two DL models can objectively partition patients into groups based on their likelihood of having sepsis and likelihood of developing shock. Second, our findings suggest that for many patients with suspected sepsis, administration of antibiotics within the rigid timeframes recommended by the SSC may not improve short term mortality. Our findings are important for several reasons. First, our data show that for patients with “probable sepsis” but who are unlikely to develop shock, mortality was similar whether antibiotics were administered before or after one hour, suggested that it is safe to defer early antibiotic administration to allow for continued work-up and evaluation. We also noted that for patients with possible sepsis (approximately 40% of the cohort), a very small percentage of patients went on to develop sepsis and had minimal change in outcome based on timing of antibiotic therapy. A sizable portion of our study population was identified as having probable sepsis and high risk for developing shock. This patient population, which had high mortality in our study, has been poorly studied and recommendations are currently lacking on how to approach management in this high-risk group. At both the development and validation sites, these patients had significantly higher risk of mortality than patients with low probability of shock, but lower mortality than patients with overt shock. Optimal care and timing of antibiotics in these patients remains unclear; however, recognition of the high-risk of these patients may inform judicious use of intensive care unit (ICU) admission, serial examinations, and other therapies (e.g., fluid resuscitation or potential corticosteroid use).

To the best of our knowledge, there are no currently validated models that can assist physicians to stratify patients objectively into SSC categories at the time of suspicion of potential sepsis. Importantly, we chose to select our time of clinical suspicion of potential sepsis which is relevant for clinicians caring for these patients and allows for meaningful interventions within a brief time. Taylor et al. conducted an observational retrospective study developed an infection prediction model that was tested amongst different clinical sites within a large health care system[14]. However, their method of assessing shock focused on the presence or absence of shock at the time of potential sepsis, which is not predictive in clinical settings. Ours is the first to demonstrate similar results via reproducible predictive DL models, applied across multiple sites at two different healthcare systems and provides an objective assessment which is applicable in a real-time clinical setting. As DL models develop and improve, these tools can be used to aid clinical judgement with broad impact on patient care and outcomes and may specifically assist with antimicrobial stewardship. Future studies will need to be completed to validate these tools at additional institutions in a variety of clinical practice settings (rural, community, county) and in different geographic locations prior to widespread use.

In 2021, the SSC updated recommendations regarding timing of antibiotic administration based on presence of shock and the likelihood of sepsis[3]. The strongest data supporting administration of empiric antibiotics comes from patients with septic shock [19–21]. On the other hand, various professional societies have expressed concerns about rigid time frames for initial antimicrobial therapy in patients with possible sepsis who are not in shock, citing concerns of lack of data supporting benefit for 1-hour time from for antibiotics, increased antibiotic resistance, collateral damage to the host, and development of nosocomial infections[7,8],[22]. Our findings suggest predictive models can be used to partition patients into risk groups which may inform treatment for those patients with probable sepsis and unlikely to develop without increased risk of death from antibiotic administration outside of the 1-hour recommended timeframe recommended by the SSC. We recognize our findings are associations and unmeasured confounders may impact the mortality findings as a function of timing of antibiotic administration. However, our data confirm the recent findings of Taylor et al. which found low mortality in sepsis patients without shock with longer times to antibiotic therapy (mean of 3.2 and 5.5 hours for patients with probable and potential sepsis, respectively)[14]. Additionally, a recent randomized trial conducted in Europe did not find a mortality benefit to the completion of a 1-hour bundle (mean time to antibiotics 40 minutes) compared to a more lenient approach (mean time to antibiotics 113 minutes) in patients with suspected sepsis in the ED [23].

Approximately 40% of patients in our cohort were partitioned into Group 1: shock unlikely and sepsis possible. This finding is important as a minority of these patients (12.9% and 14.7% at the development and validation sites, respectively) went on to meet the definition of sepsis. However, 75% of patients in this group at the development site and 88.6% at the validation site received antibiotics, which speaks to possible scale of overtreatment with antibiotics. Additional diagnostic testing and evaluation potentially outside the 3-hour window recommended by the SSC and SEP-1 may be safe for these patients. Future investigations are needed to determine further how the probability of sepsis should impact timing of antibiotic recommendations, as the SSC provided a strong recommendation for antibiotic therapy within 3 hours but noted “very low level of evidence” in their 2021 guidelines [3]. Likewise, SEP-1 does not allow providers to deviate from the 3-hour time to administer antibiotics for patients with possible severe sepsis and septic shock [6].

Prior investigations have highlighted risk factors for the development of delayed septic shock in ED patients (e.g., advanced age, sex, certain comorbidities, hyperlactatemia), yet these are not actionable[17,18,24,25]. Importantly, these patients have high mortality rates, despite a less severe initial presentation that patients with septic shock present at or near the time of ED triage[17,25]. In our analysis, we noted that Shock-Net was able to identify accurately patients who would later develop septic shock. We also found that the mortality of patients in the sepsis probable and shock likely cohort was significantly higher than patients where shock was unlikely. Our sensitivity analysis, in which we excluded patients who developed shock within 3 hours of ED triage, produced similar trends in mortality and time to antibiotics at the development and validation sites. Early identification of these patients (ones that clinicians would arguably need less aid to identify and stratify without a predictive model) may help inform care through increased surveillance or inform additional therapeutics (e.g., thoughtful decisions regarding fluid administration, reassessment of appropriateness of antibiotics, source control) may improve patient-centered outcomes in this high-risk population. Interestingly, we also noted that mortality rates were highest in these patients who received antibiotics within 1 hour of triage or time of clinical suspicion of potential sepsis compared to those who received antibiotics within 1–3 hours or greater than 3 hours. One explanation is that this group (patients who received antibiotics within 1 hour) simply represent a population with a higher severity of illness than those who received antibiotics after one hour.

## Limitations

This investigation has several limitations. First, our data were curated from two academic medical centers and although our patient population is diverse, our findings may not be generalizable to all settings. Second, although we used DL models with prospectively applied data, we did not assess how physicians interact with these models and how use of these models impacts antimicrobial prescribing and patient-centered outcomes. Our definitions of potential sepsis, sepsis and shock were selected based on our prior work; however, use of different constructs of these terms may impact results. Additionally, different input features may impact our predictive models; however, we selected commonly used input features which are currently used in clinical practice at our health system. The number of patients with shock, possible sepsis is smaller compared to the other groups and this trend was observed at both the development and training site. The size of this group may make it challenging to appreciate trends and could reflect the patient population, training model, and prevalence of distributive shock across emergency departments. Finally, our investigation was retrospective, and we described associations between timing of antibiotics and short term mortality. Our findings should not be used to infer causality.

## Conclusions

We demonstrate that two DL models can stratify patients with potential sepsis into partitions based on probability of sepsis and shock development using patient data from two academic health systems. Our data suggest that administration of antibiotics after one hour may be safe in patients with probable sepsis and low likelihood of shock, however, we did not assess causality. Mortality was low in the small number of patients with possible sepsis and a low likelihood of shock who later developed sepsis~~.~~, even when receiving antibiotics after the recommended 3-hour timeframe. Further prospective studies in diverse practice settings are required to validate these findings.

## Declarations

**Ethics approval and consent to participate:** Institutional review board approval with waiver of informed consent was obtained.

**Consent for publication:** N/A

**Availability of data and materials:** The data that support the findings of this study are available from Gabriel Wardi, upon reasonable request.

**Competing interests:** S.N., A. B., and A.M. are cofounders, advisors, and hold equity in Clairyon Inc. (formerly Healcisio Inc.), a medical software startup. The terms of this arrangement have been reviewed and approved by the UC San Diego in accordance with its conflict-of-interest policies. S.N. and A.M. report income from Powell Mansfield, a startup focused on diagnosis of sleep disordered breathing. A.M. additionally reports income related to medical education from Livanova, Eli Lilly, and Zoll. The remaining authors declare no competing interests.

**Funding:** G.W. and J.F. are supported by the National Foundation of Emergency Medicine. G.W is supported by the National Institutes of Health (#K23GM146092). B.G. and A.B. are funded by the National Library of Medicine (#2T15LM011271-11). S.N. acknowledges grant funding from the U.S. National Library of Medicine (award number R01LM013998), the U.S. National Institute of General Medical Sciences (award number R35GM143121), and the U.S. National Science Foundation (award number 2405974). S.N., A.M., and A.B., acknowledges grant funding from the U.S. National Institute of Allergy and Infectious Diseases (award number R42AI177108). S.N., J.F. and K.Z. acknowledge support through the Multicampus Research Programs and Initiatives (MRPI) grant from the University of California (award number M25PR9018).

**Authors' contributions:** Conception/Design: Acquisition of data: SN, GW, JF, AM, KZ, XL, AB; Analysis: BG, SN, GW, JF, AD; Drafting of manuscript: BG, GW, JF, SN; Critical Revisions: BG, GW, JF, SN, AM.

**Acknowledgements:** Supreeth Shashikumar PhD provided assisted with the development of the Shock-Net code.

## Tables

**Table 1:** Development Site ED Stratification, Mortality, and Antibiotic Timing Among Patients Treated with Suspected Sepsis Infection Between Jan 2016 and Dec 2023.

| Variables | Group 1<br><br>Shock Likely to Develop and Sepsis Probable<br><br>(n=3651) | Group 2<br><br>Shock Likely to Develop and Sepsis Possible<br><br>(n=124) | Group 3<br><br>Shock Unlikely to Develop and Sepsis Probable<br><br>(n=14774) | Group 4<br><br>Shock Unlikely to Develop and Sepsis Possible<br><br>(n=11241) |
|---|---|---|---|---|
| **Patient Characteristics** | | | | |
| Age Yrs [IQR] | 66 [55 - 78] | 66 [48 - 78] | 59 [42 - 73] | 55 [37 - 71] |
| Female % (n) | 43.5 (310/712) | 32.7 (17/52) | 43.6 (724/1659) | 47.6 (929/1950) |
| Black Race % (n) | 1.4 (10/712) | 3.8 (2/52) | 2.4 (39/1659) | 3.5 (68/1950) |
| White Race % (n) | 42.8 (305/712) | 44.2 (23/52) | 44.7 (741/1659) | 45.9 (896/1950) |
| Asian Race % (n) | 24.2 (172/712) | 26.9 (14/52) | 20.4 (338/1659) | 17.7 (345/1950) |
| Charlson Comorbidity Index [IQR] | 1 [0 - 3] | 0 [0 - 2] | 1 [0 - 3] | 1 [0 - 2] |
| SOFA [IQR] | 3 [1 - 5] | 2 [1 - 5] | 1 [0 - 2] | 0 [0 - 1] |
| Confirmed Sepsis[a], % (n) | 73.5 (2685/3651) | 59.7 (74/124) | 36.0 (5320/14774) | 14.7 (1655/11241) |
| Developed Shock, % (n) | 44.1 (1611/3651) | 36.3 (45/124) | 6.0 (888/14774) | 1.6 (182/11241) |
| **Short term Mortality[b]** | | | | |
| Overall, % (n) | 23.1 (845/3651) | 17.7 (22/124) | 5.0 (742/14774) | 1.9 (216/11241) |

[a]Patients who met Sepsis 3 criteria during hospitalization. [b] Composite of in-hospital mortality and discharge to hospice.

**Table 2:** Validation Site ED Stratification, Mortality, and Antibiotic Timing Among Patients Treated with Suspected Sepsis Infection Between Jan 2023 and Oct 2024.

| Variables | Group 1 Shock Likely to Develop and Sepsis Probable (n=712) | Group 2 Shock Likely to Develop and Sepsis Possible (n=53) | Group 3 Shock Unlikely to Develop and Sepsis Probable (n=1659) | Group 4 Shock Unlikely to Develop and Sepsis Possible (n=1950) |
|---|---|---|---|---|
| **Patient Characteristics** | | | | |
| Age Yrs [IQR] | 66 [55 - 78] | 66 [48 - 78] | 59 [42 - 73] | 55 [37 - 71] |
| Female % (n) | 43.5 (310/712) | 32.7 (17/52) | 43.6 (724/1659) | 47.6 (929/1950) |
| Black Race % (n) | 1.4 (10/712) | 3.8 (2/52) | 2.4 (39/1659) | 3.5 (68/1950) |
| White Race % (n) | 42.8 (305/712) | 44.2 (23/52) | 44.7 (741/1659) | 45.9 (896/1950) |
| Asian Race % (n) | 24.2 (172/712) | 26.9 (14/52) | 20.4 (338/1659) | 17.7 (345/1950) |
| Charlson Comorbidity Index [IQR] | 1 [0 - 3] | 0 [0 - 2] | 1 [0 - 3] | 1 [0 - 2] |
| SOFA [IQR] | 3 [1 - 5] | 2 [1 - 5] | 1 [0 - 2] | 0 [0 - 1] |
| Confirmed Sepsis[a], % (n) | 71.1 (506/712) | 42.3 (22/52) | 34.5 (572/1659) | 12.9 (251/1950) |
| Developed Shock, % (n) | 37.1 (264/712) | 19.2 (10/52) | 5.1 (85/1659) | 1.2 (24/1950) |
| **Short term Mortality[d]** | | | | |
| Overall, % (n) | 28.5 (203/712) | 15.4 (8/52) | 8.3 (137/1659) | 2.9 (57/1950) |

[a] Patients who met Sepsis 3 criteria during hospitalization. [b] Median time in hours. [c] T0 = time of clinical suspicion of potential sepsis. [d] Composite of in-hospital mortality and discharge to hospice.

Table 3: Short Term Mortality by Sepsis Risk Group, Antibiotic Timing and Study Site

| Short Term Mortality, % (n) | Antibiotic Timing (From Triage) <1 hour | 1-3 hours | >3 hours | p-value 1[a] | p-value 2[b] |
|---|---|---|---|---|---|
| **Group 1 [Shock Likely to Develop and Sepsis Probable]** | | | | | |
| **Development Site** | 25.2 (236/935) | 24.1 (397/1645) | 23.3 (212/908) | 0.789 | 0.749 |
| **Validation Site** | 31.7 (57/180) | 30.8 (92/299) | 27.4 (54/197) | 0.679 | 0.805 |
| **Group 2 [Shock Likely to Develop and Sepsis Possible]** | | | | | |
| **Development Site** | 30.0 (3/10) | 20.5 (9/44) | 17.5 (10/57) | 0.667 | 0.648 |
| **Validation Site** | 16.7 (1/6) | 22.2 (4/18) | 12.5 (3/24) | 0.500 | 0.651 |
| **Group 3 [Shock Unlikely to Develop and Sepsis Probable]** | | | | | |
| **Development Site** | 5.9 (108/1834) | 5.5 (285/5169) | 5.9 (349/5914) | 0.592 | 0.253 |
| **Validation Site** | 7.3 (15/205) | 8.7 (40/461) | 9.2 (82/893) | 0.253 | 0.292 |
| **Group 4 [Shock Unlikely to Develop and Sepsis Possible]** | | | | | |
| **Development Site** | 5.0 (20/398) | 2.5 (52/2089) | 2.4 (144/6026) | 0.999 | 0.898 |
| **Validation Site** | 6.5 (9/138) | 4.6 (13/281) | 2.7 (35/1310) | 0.975 | 0.992 |

[a]One sided Chi-squared test to determine if the mortality of those who received antibiotics within an hour of triage in each risk group were significantly lower than the other patients within the risk group

[b]One-sided Chi-squared test to determine if those who received antibiotics after 3 hours of triage had significantly higher mortality compared to the other patients within the risk group

Table 4: Short Term Mortality by Sepsis Risk Group, Antibiotic Timing and Study Site, Excluding Patients Who Develop Shock within 3 Hours of Triage

| | Antibiotic Timing (From Triage) | | | | |
|---|---|---|---|---|---|
| Short Term Mortality, % (n) | <1 hour | 1-3 hours | >3 hours | p-value 1[a] | p-value 2[b] |
| **Group 1 [Shock Likely to Develop and Sepsis Probable)** | | | | | |
| **Development Site** | 21.4 (120/56) | 18.7 (211/1128) | 18.7 (132/707) | 0.915 | 0.682 |
| **Validation Site** | 30.5 (36/118) | 26.4 (55/208) | 22.1 (36/163) | 0.879 | 0.899 |
| **Group 2 [Shock Likely to Develop and Sepsis Possible]** | | | | | |
| **Development Site** | 33.3 (2/6) | 16.7 (5/30) | 13.3 (6/45) | 0.733 | 0.670 |
| **Validation Site** | 0.0 (0/5) | 7.1 (1/14) | 13.0 (3/23) | 0.500 | 0.372 |
| **Group 3 [Shock Unlikely to Develop and Sepsis Probable]** | | | | | |
| **Development Site** | 5.4 (94/174) | 5.0 (251/5007) | 5.6 (328/5822) | 0.507 | 0.104 |
| **Validation Site** | 7.1 (14/198) | 7.6 (34/450) | 9.1 (80/882) | 0.285 | 0.143 |
| **Group 4 [Shock Unlikely to Develop and Sepsis Possible]** | | | | | |
| **Development Site** | 5.1 (20/395) | 2.4 (49/2062) | 2.3 (137/5989) | 1.00 | 0.908 |
| **Validation Site** | 5.8 (8/137) | 4.3 (12/279) | 2.6 (34/1305) | 0.949 | 0.981 |

[a]One sided Chi-squared test to determine if the mortality of those who received antibiotics within an hour of triage in each risk group were significantly lower than the other patients within the risk group
[b]One-sided Chi-squared test to determine if those who received antibiotics after 3 hours of triage had significantly higher mortality compared to the other patients within the risk group

## Figures

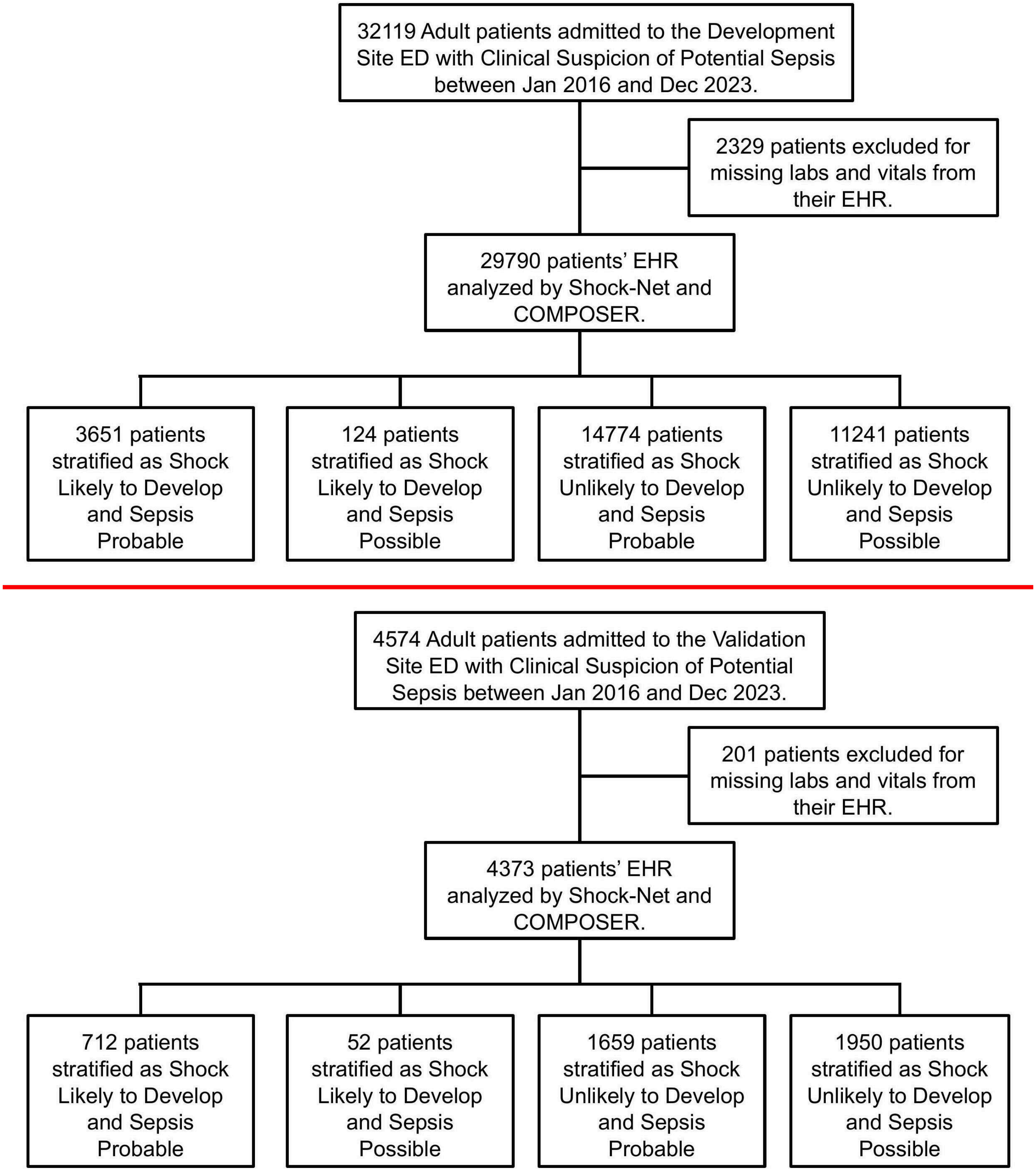

**Figure 1**

Patient inclusion and categorization for the Development Site (Top), and the Validation Site (Bottom)

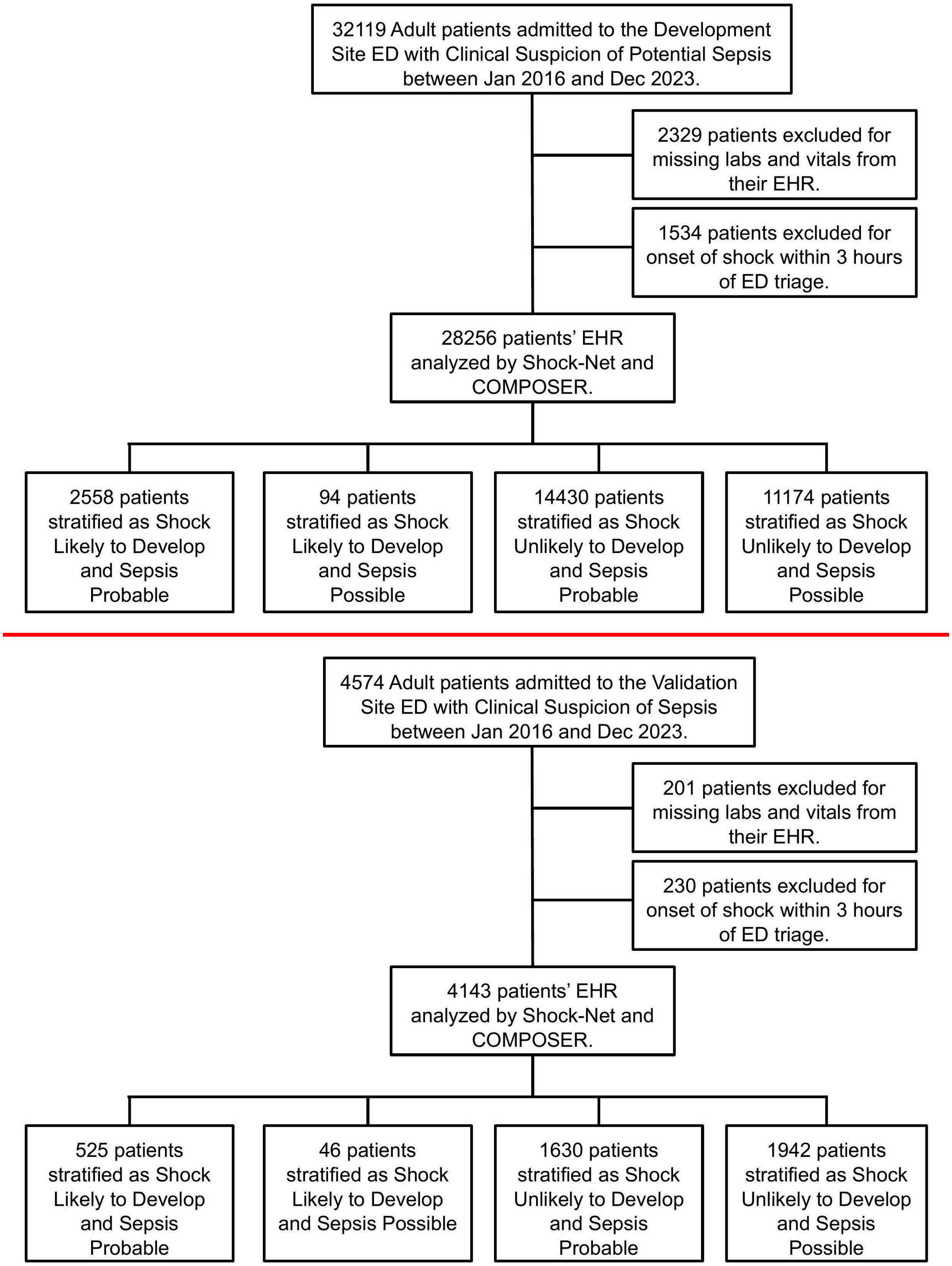

### Figure 2

Flow diagram showing patient inclusion and categorization for the Development Site ER (Top), and the Validation Site ER (Bottom) for study where we exclude patients who develop shock within 3 hours of ED triage

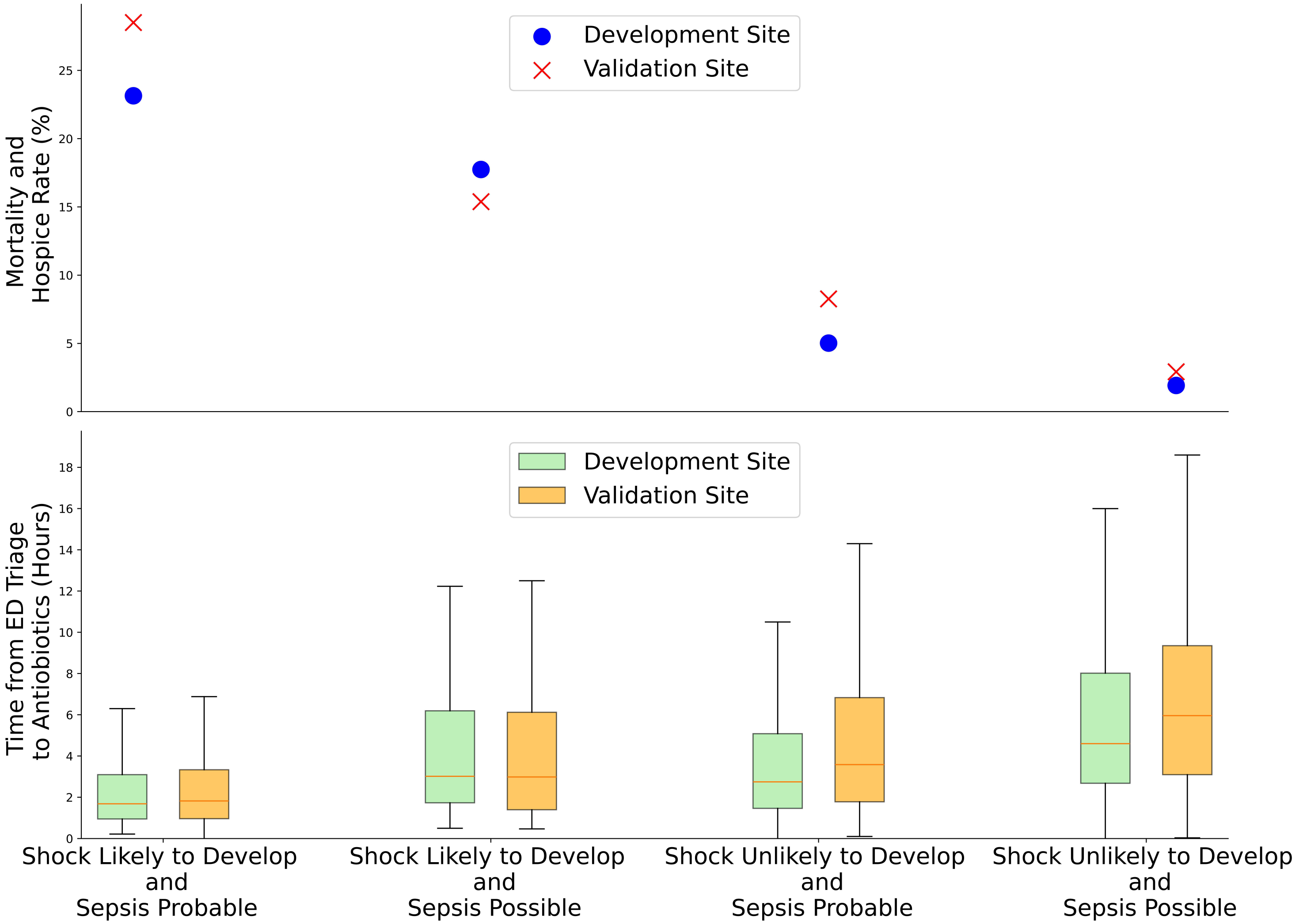

### Figure 3

Comparison of mean mortality and time to antibiotics (IQR) between the Development Site and Validation Site. Short-term mortality is the composite outcome of in-hospital mortality and transition to hospice

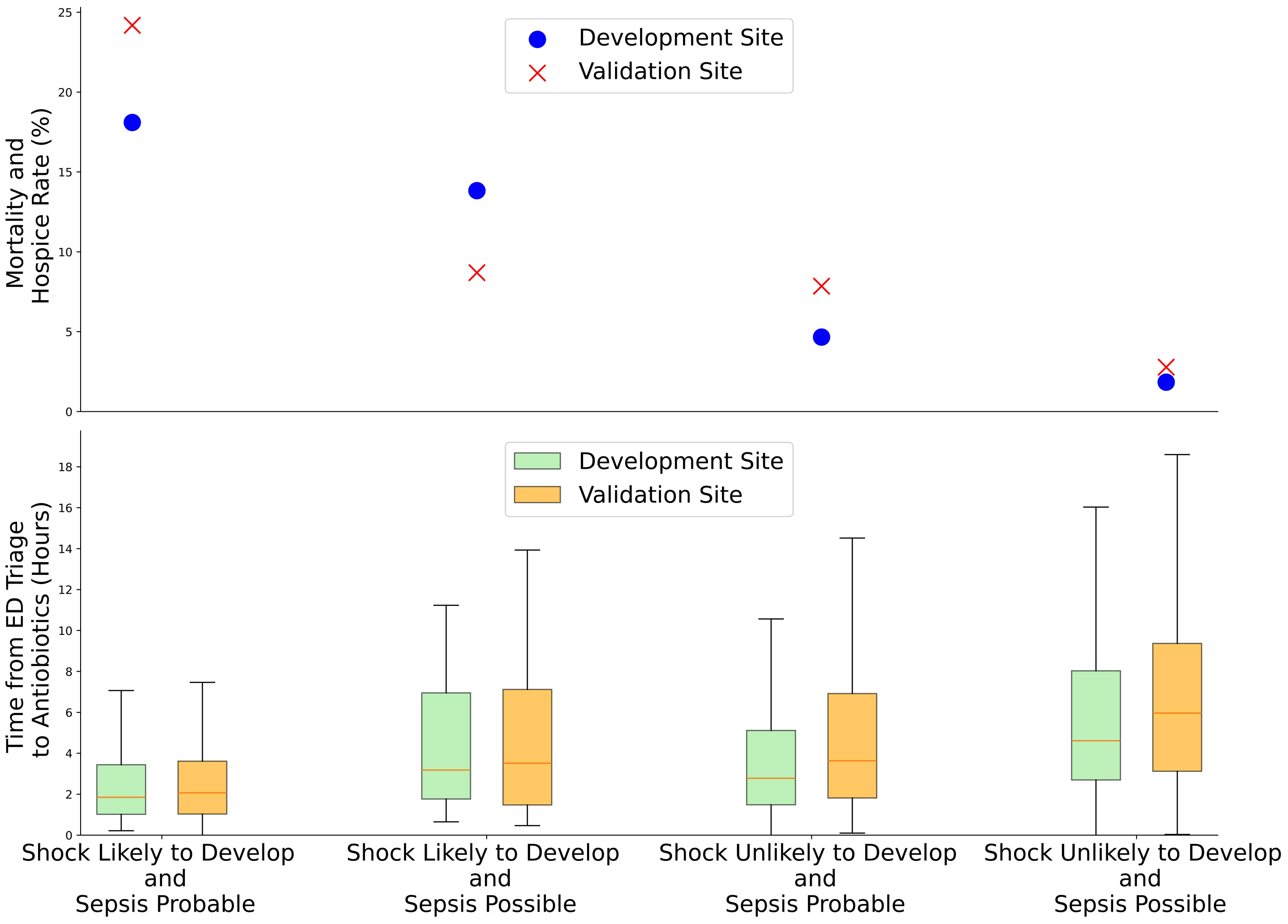

**Figure 4**

Comparison of mean mortality and time to antibiotics (IQR) between Development Site and Validation Site Excluding Patients Who Developed Shock within the first 3 hours of triage

## Supplementary Files

This is a list of supplementary files associated with this preprint. Click to download.

- CCMMortalityABXsupplementforresubmissionafterCCreviewerfeedback.docx
- SupplementaryFigure1.jpg
- SupplementaryFigure2.jpg
- SupplementaryFigure3.jpg
- SupplementaryFigure4.jpg
